# Supplementary material for: Insights from a genome-wide truth set of tandem repeat variation
Source: bioRxiv. 2023 May 8:2023.05.05.539588. Preprint. [Version 1] doi: 10.1101/2023.05.05.539588 (PMC10197592; doi:10.1101/2023.05.05.539588)
Supplement: Supplement 1 [file NIHPP2023.05.05.539588v1-supplement-1.pdf]

bioRxiv preprint doi: https://doi.org/10.1101/2023.05.05.539588; this version posted May 8, 2023. The copyright holder for this preprint (which was not certified by peer review) is the author/funder, who has granted bioRxiv a license to display the preprint in perpetuity. It is made available under aCC-BY 4.0 International license.

Supplementary Figure 1

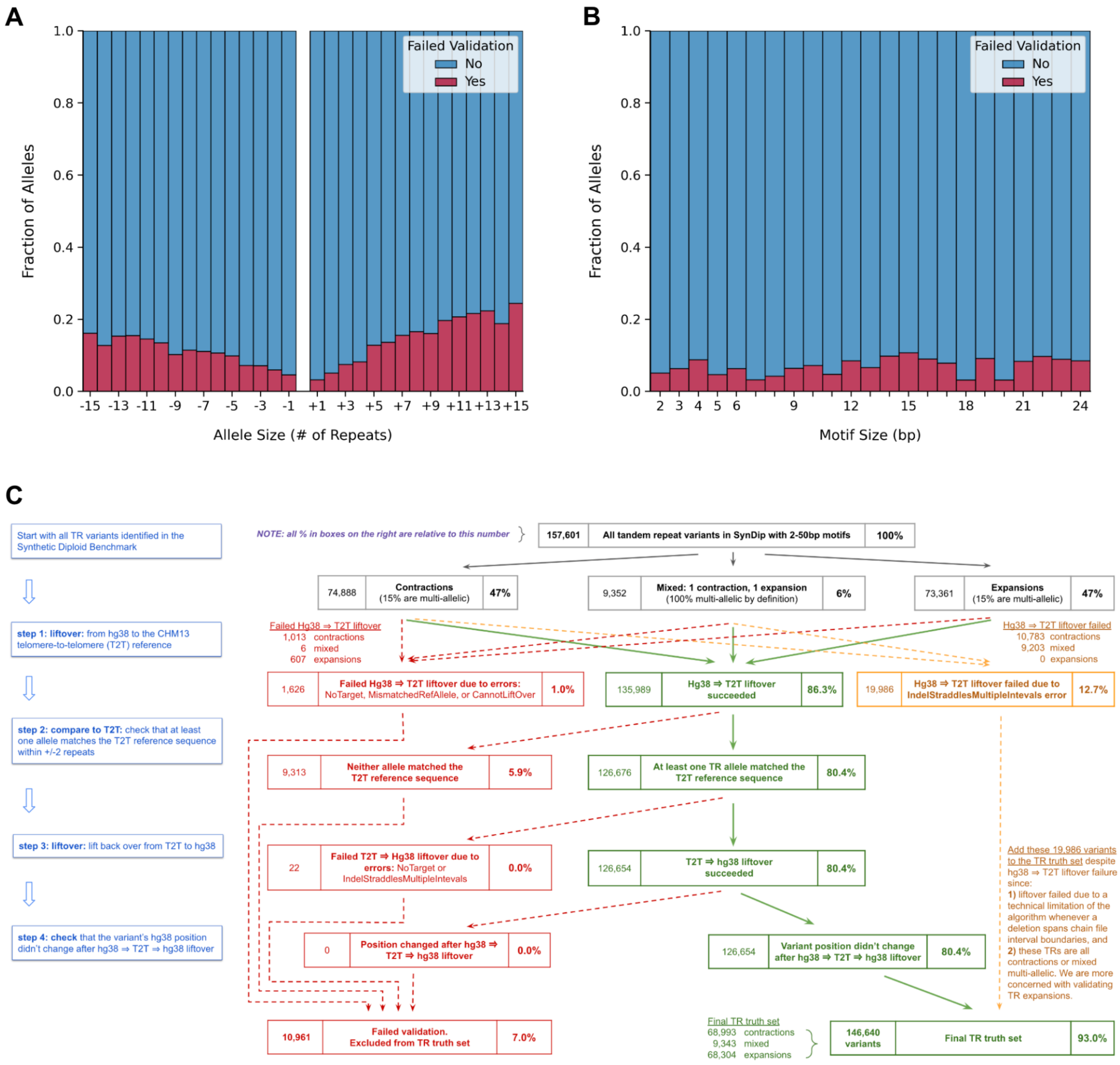

Supplementary Figure 1: Validation of TR variants via liftover to the telomere-to-telomere (T2T) reference genome

A. Distribution of allele sizes for loci that failed validation. The x-axis represents SynDip allele sizes relative to the hg38 reference. Red represents the fraction (y-axis) of alleles that occurred at loci which failed validation.

bioRxiv preprint doi: https://doi.org/10.1101/2023.05.05.539588; this version posted May 8, 2023. The copyright holder for this preprint (which was not certified by peer review) is the author/funder, who has granted bioRxiv a license to display the preprint in perpetuity. It is made available under aCC-BY 4.0 International license.

B. Distribution of motif sizes for loci that failed validation. The x axis represents TR motif sizes ranging from 2 to 24bp. The y axis shows, for each motif size, the fraction of alleles that occurred at loci which failed validation.

C. Validation procedure flowchart and statistics. This flowchart shows the 4 steps (blue) used to validate TR truth set variants via comparison to the T2T reference sequence, as well as the number of variants that passed (green) or failed (red) each step. It also shows the contractions and mixed multi-allelic variants that could not be validated (orange) due to technical limitations of the liftover algorithm. In the end, 10,961 (7%) of TR variants failed validation.

bioRxiv preprint doi: https://doi.org/10.1101/2023.05.05.539588; this version posted May 8, 2023. The copyright holder for this preprint (which was not certified by peer review) is the author/funder, who has granted bioRxiv a license to display the preprint in perpetuity. It is made available under aCC-BY 4.0 International license.

Supplementary Figure 2

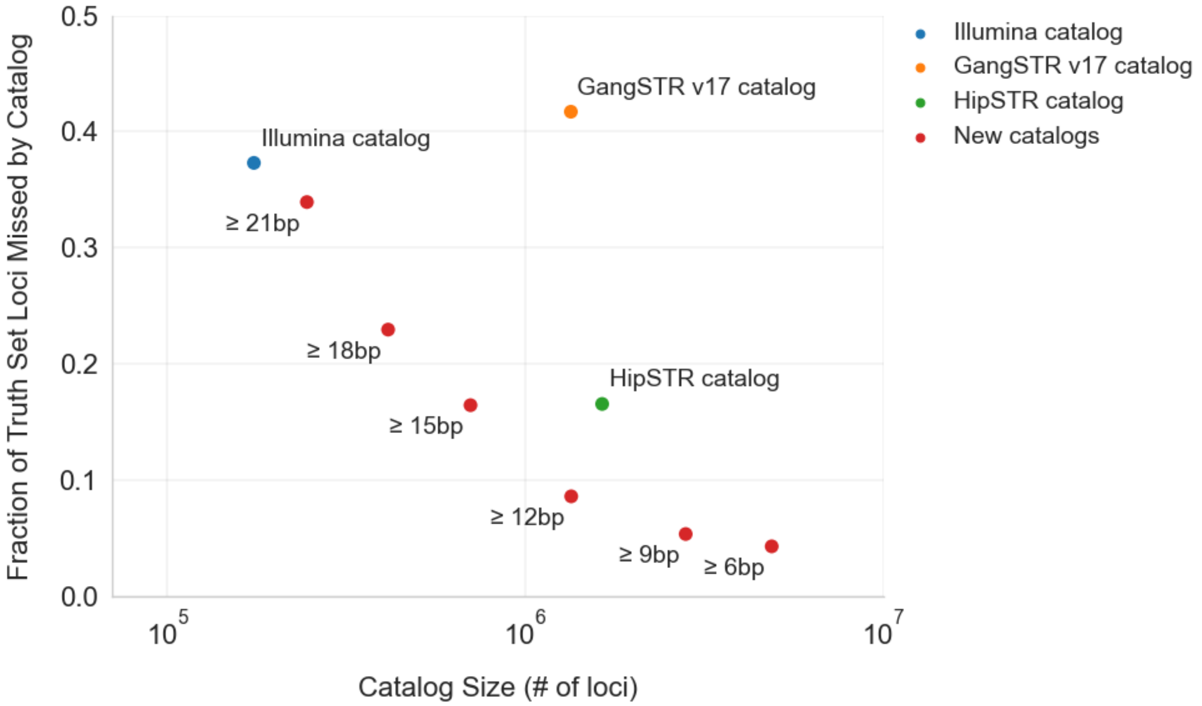

Supplementary Figure 2: Catalogs by size and overlap with truth set loci

The scatter plot includes a dot for each of 3 publicly available TR catalogs (blue, orange, green), as well as 7 new catalogs (red) that we generated by using TRF. Each new catalog contains pure repeats in hg38 that span no less than the labeled minimum threshold of base pairs. The x axis shows the number of loci in each catalog and the y axis shows what fraction of the TR truth set is missed by this catalog. Missed loci are defined as truth set loci that don't overlap any of the repeat loci in a catalog.

bioRxiv preprint doi: https://doi.org/10.1101/2023.05.05.539588; this version posted May 8, 2023. The copyright holder for this preprint (which was not certified by peer review) is the author/funder, who has granted bioRxiv a license to display the preprint in perpetuity. It is made available under aCC-BY 4.0 International license.

Supplementary Figure 3

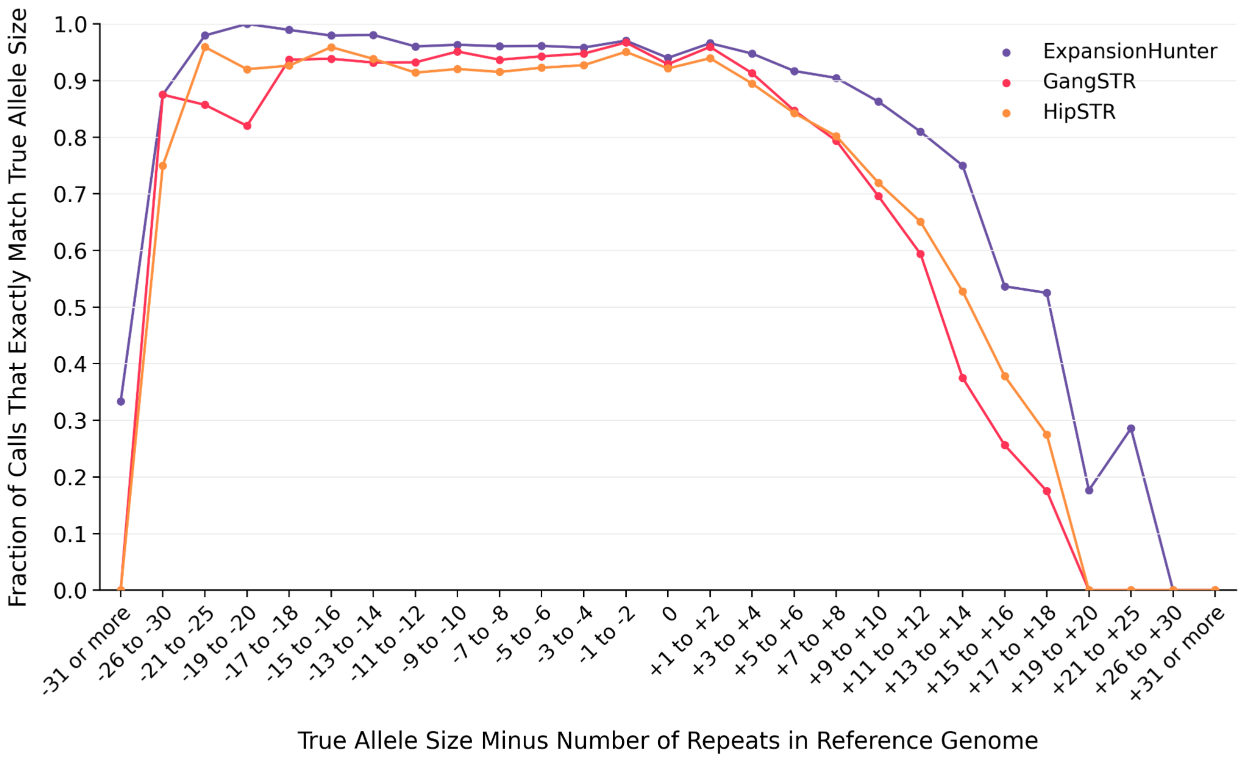

Supplementary Figure 3: Strict accuracy after excluding no-call loci

Like Figure 4b, this plot shows the fraction of alleles that each tool got exactly right (y axis) across different true allele size bins (x axis). Here, the plot excludes all loci where one or more tools did not produce a genotype instead of treating them as equivalent to incorrect calls.

bioRxiv preprint doi: https://doi.org/10.1101/2023.05.05.539588; this version posted May 8, 2023. The copyright holder for this preprint (which was not certified by peer review) is the author/funder, who has granted bioRxiv a license to display the preprint in perpetuity. It is made available under aCC-BY 4.0 International license.

Supplementary Figure 4

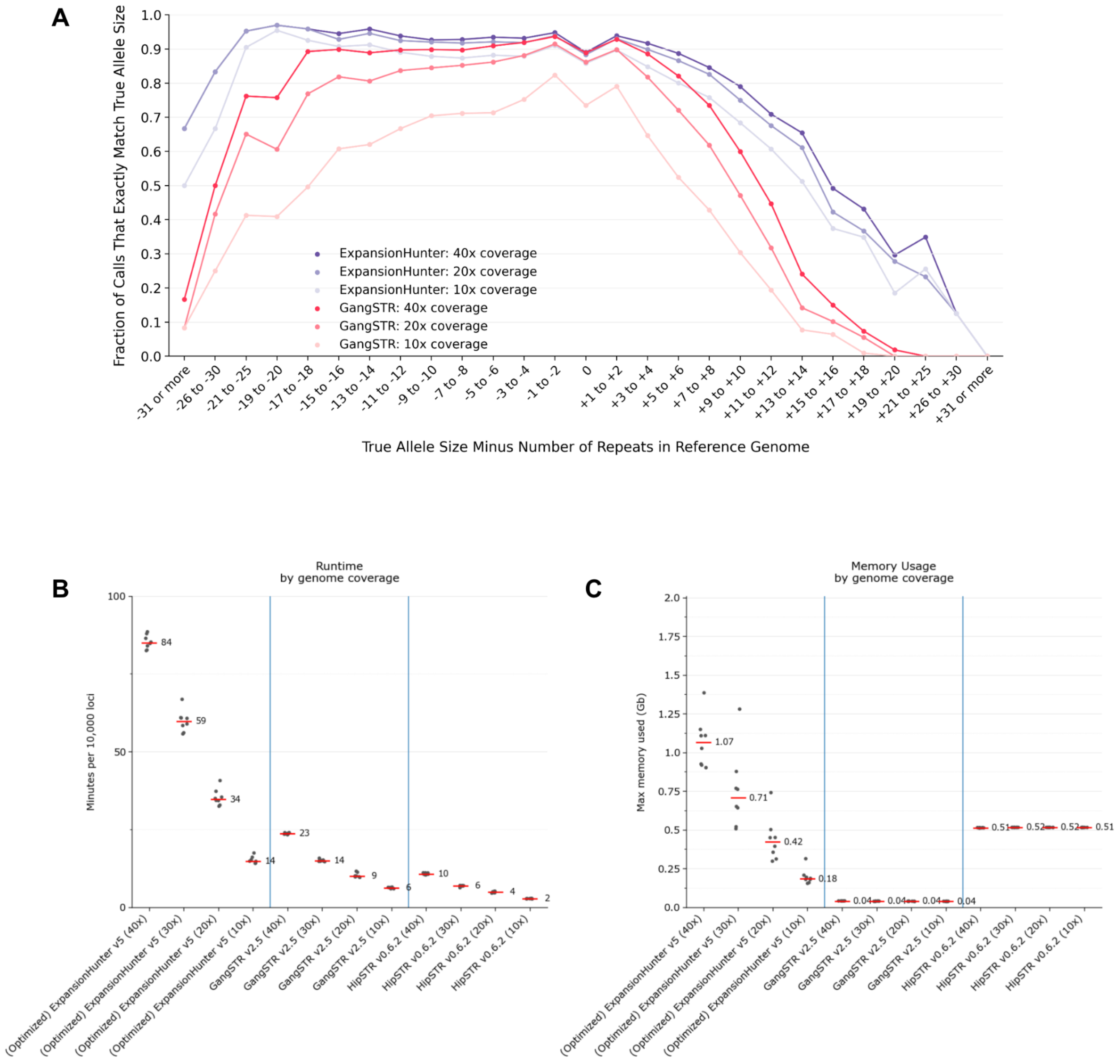

Supplementary Figure 4: Tool accuracy, runtimes and memory use by read depth

A. ExpansionHunter and GangSTR accuracy at different read depths. This plot compares strict accuracy for each tool across 3 different coverage levels: 40x, 20x, 10x.

B. Tool runtimes by read depth. For ExpansionHunter, GangSTR, and HipSTR, tool runtime in minutes per 10k loci (y-axis) is proportional to the depth of coverage of the input sample (x-axis) - as shown for coverage levels: 40x, 30x, 20x, 10x.

bioRxiv preprint doi: https://doi.org/10.1101/2023.05.05.539588; this version posted May 8, 2023. The copyright holder for this preprint (which was not certified by peer review) is the author/funder, who has granted bioRxiv a license to display the preprint in perpetuity. It is made available under aCC-BY 4.0 International license.

C. Tool memory usage by coverage. The optimized version of ExpansionHunter uses read caching to speed up execution, and so has a higher peak memory usage (y-axis) at higher coverage levels (x-axis). Other tools' memory usage doesn't vary with depth of coverage.

bioRxiv preprint doi: https://doi.org/10.1101/2023.05.05.539588; this version posted May 8, 2023. The copyright holder for this preprint (which was not certified by peer review) is the author/funder, who has granted bioRxiv a license to display the preprint in perpetuity. It is made available under aCC-BY 4.0 International license.

Supplementary Figure 5

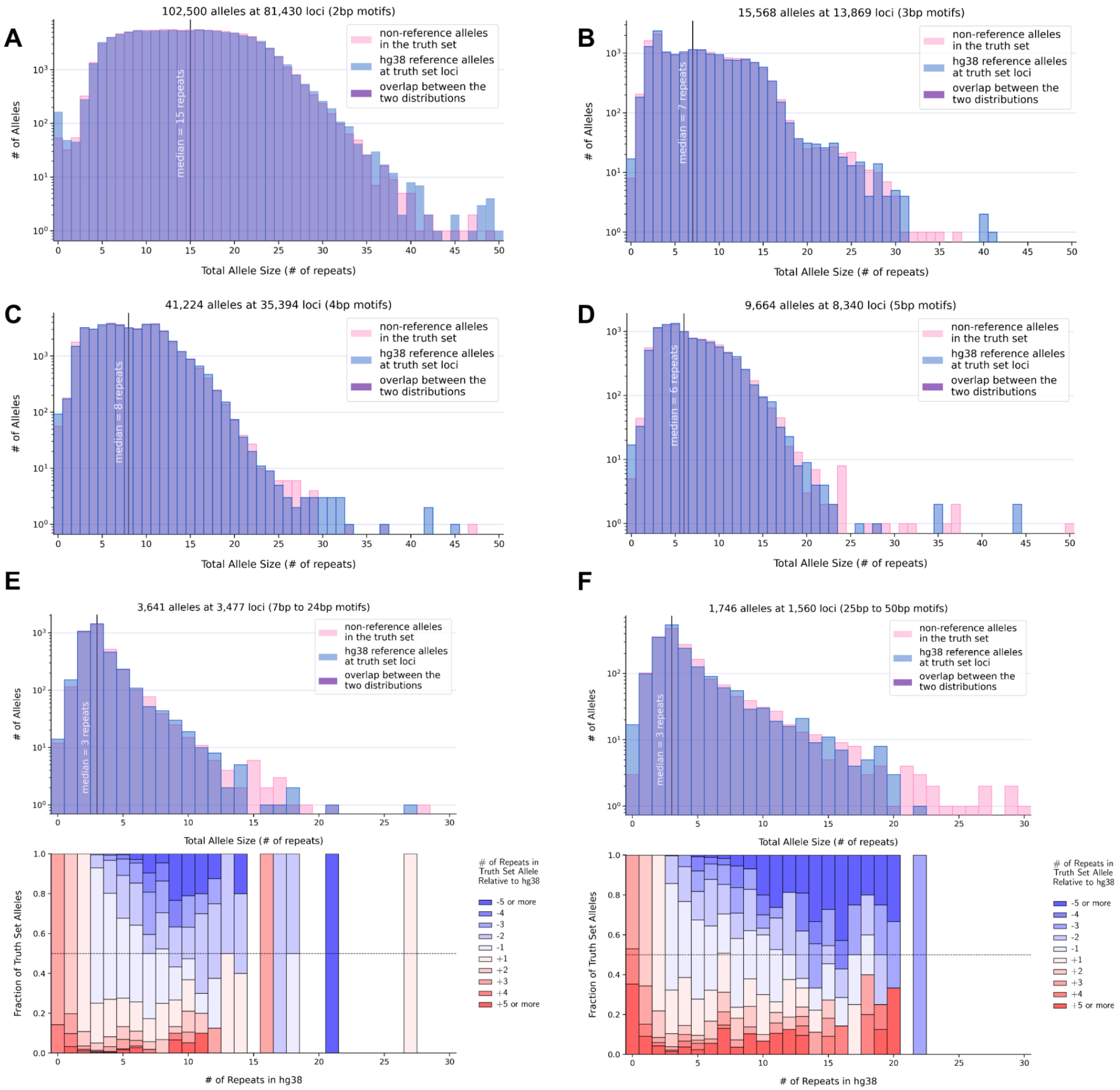

Supplementary Figure 5: TR allele and motif sizes compared to repeat sequences in hg38 for 7-24bp and 25-50bp motifs

A. Truth set allele sizes vs reference allele sizes with 2bp motifs. The x axis shows allele sizes in terms of numbers of repeats. The y axis counts how many alleles of the

bioRxiv preprint doi: https://doi.org/10.1101/2023.05.05.539588; this version posted May 8, 2023. The copyright holder for this preprint (which was not certified by peer review) is the author/funder, who has granted bioRxiv a license to display the preprint in perpetuity. It is made available under aCC-BY 4.0 International license.

given size (x axis) were found among truth set non-reference alleles (pink) or among hg38 reference alleles at truth set loci (blue). The two distributions - one pink, one blue - are plotted on top of each other using semi-transparent colors so that their overlap appears purple. The vertical line at x = 15 indicates the median number of repeats, which has the same value for both distributions. For the blue distribution, the reference allele size is counted twice at multi-allelic loci so that the two distributions have the same number of counts.

B. Truth set allele sizes vs reference allele sizes with 3bp motifs. Same as panel A but for 3bp motifs. The median is at x = 7.

C. Truth set allele sizes vs reference allele sizes with 4bp motifs. Same as panel A but for 4bp motifs. The median is at x = 8.

D. Truth set allele sizes vs reference allele sizes with 5bp motifs. Same as panel A but for 5bp motifs. The median is at x = 6.

E. Truth set allele sizes vs reference allele sizes with 7-24bp motifs. Same as panel A but for 7-24bp motifs. The median is at x = 3. Expansions and contractions in the truth set are shown in the lower panel using colors as in **Figure 3B**.

F. Truth set allele sizes vs reference allele sizes with 25-50bp motifs. Same as panel A but for 25-50bp motifs. The median is at x = 3. Expansions and contractions in the truth set are shown in the lower panel using colors as in **Figure 3B**.
